# A Review of Heartbeat Detection Systems for Automotive Applications

**DOI:** 10.3390/s21186112

**Published:** 2021-09-12

**Authors:** Toshiya Arakawa

**Affiliations:** Department of Information Technology and Media Design, Nippon Institute of Technology, Miyashiro-machi, Saitama 345-0826, Japan; arakawa.toshiya@nit.ac.jp; Tel.: +81-480-34-4111

**Keywords:** heartbeat monitoring system, driver, smartwatch, in-vehicle

## Abstract

Many accidents are caused by sudden changes in the physical conditions of professional drivers. Therefore, it is quite important that the driver monitoring system must not restrict or interfere with the driver’s action. Applications that can measure a driver’s heartbeat without restricting the driver’s action are currently under development. In this review, examples of heartbeat-monitoring systems are discussed. In particular, methods for measuring the heartbeat through sensing devices of a wearable-type, such as wristwatch-type, ring-type, and shirt-type devices, as well as through devices of a nonwearable type, such as steering-type, seat-type, and other types of devices, are discussed. The emergence of wearable devices such as the Apple Watch is considered a turning point in the application of driver-monitoring systems. The problems associated with current smartwatch- and smartphone-based systems are discussed, as are the barriers to their practical use in vehicles. We conclude that, for the time being, detection methods using in-vehicle devices and in-vehicle cameras are expected to remain dominant, while devices that can detect health conditions and abnormalities simply by driving as usual are expected to emerge as future applications.

## 1. Introduction

Many accidents are caused by sudden changes in the physical conditions of professional drivers. A report shows that, compared to other leading causes of death, road fatalities are lower, but still quite substantial, the researchers say. Nationally, fatalities from road crashes per 100,000 population is 10.9, compared to 34.4 for Alzheimer’s, 43.7 for stroke, 48.2 for lung disease, 185.4 from cancer and 197.2 from heart disease [1]. Therefore, traffic accidents caused by heart disease seem to be an important problem. For example, in Mississippi, a school bus crashed, and several children were injured after the bus driver suffered “sudden cardiac death” [2,3,4,5,6]. In 2016, in the downtown area of Kita-ku, Osaka City, Japan, a car went out of control and hit a pedestrian crossing an intersection with the result that two people, including the driver, died, and eight other adults received light to severe injuries [7,8,9]. It was stated that the driver had lost consciousness at the time, and staff at the facility management company of the driver said he had not disclosed any ongoing medical conditions and appeared normal at a meeting before departing on business in a company-owned car [9]. Systems have been developed to implement an emergency procedure for taking driving control away from a driver when sudden changes occur in the driver’s physical condition, such that the driver loses control of the vehicle [10,11]. In some cases, vehicles are equipped with such an emergency system [12,13,14,15,16,17,18,19]. For example, an emergency system of Mercedes-Benz has several steps. When the driver is no longer interacting with the steering wheel, the system flashes a light and sounds a tone to alert the driver to return his/her hands to the steering wheel. If the driver still does not respond, the system applies the brakes. As it slows, the system maintains the lane in which the vehicle is already traveling [18].

However, it is desirable to be able to detect whether a driver’s driving competency is deteriorating so that the driver does not experience a sudden change in physical condition while driving. In Japan, the Ministry of Land, Infrastructure, Transport, and Tourism published a “health management manual for drivers of commercial vehicles” in 2010 (revised in 2014) [20]. The manual addresses the health management of the commercial drivers themselves, in the context of overall business management. It states that devices and software to check a driver’s health are required as part of daily management procedures. Consequently, it is expected that driver-state monitoring systems will be developed in the future. Such driver monitoring systems are required not only to monitor drivers’ physical health but also to take control of the driving of the vehicle, according to the Society of Automotive Engineers (SAE)’s standards for autonomous driving level 3 [21]. Driver monitoring systems are characterized by the acquisition of the driver’s biometric information to understand the driver’s condition and reflect the results of vehicle control.

**Figure 1:**
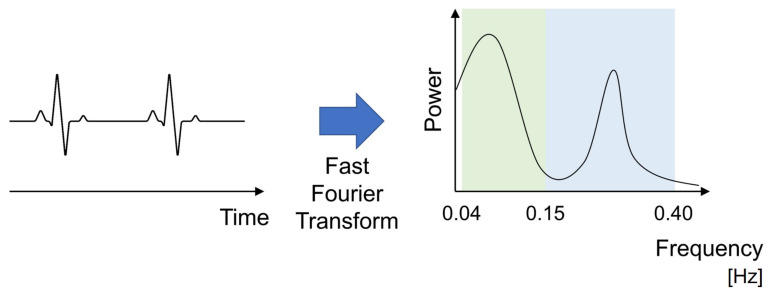
The relation of heartbeat, HF and LF.

Biometric information includes “in vivo information” such as an electrocardiogram (ECG), electrodermal activity (EDA), blood pressure levels, and visceral fat levels; and “ex vivo information” such as exercise levels, sleep patterns, and diet, and it is important to evaluate, associate, and analyze these data correctly. The instantaneous heartbeat, which is calculated from the ECG, is an especially important information datum. In the normal state, the instantaneous heart rate fluctuates within a certain range of variation. This range is called heart rate variability (HRV). Analysis of HRV allows the evaluation of the tension of the autonomic nervous system and the detection of angina and ischemic heart disease to categorize various diseases [22]. HRV reflects the balance between sympathetic nerves and parasympathetic nerves [23]. In waveforms obtained by spectral analysis of HRV, the Mayer wave (low frequency (LF) components, generally between 0.04 and 0.15 Hz [24]), which is a signal source of blood pressure variability with a period of about 10 s, is a sign of increased sympathetic nerve activity or decreased vagal nerve activity, and is said to indicate driver arousal or attention. In contrast, high-frequency (HF) components (generally between 0.15 and 0.40 Hz [24]) signify decreased sympathetic nerve activity, increased parasympathetic nerve activity, and vagal nerve activity and are said to indicate fatigue and sleep [25]. In addition to the analysis of heart rate intervals, LF/HF is a commonly used indicator because it can evaluate the driver’s attention and arousal state by assessing the LF and HF components—specifically, the ratio of LF to HF. Here, LF and HF are generally calculated by the power spectrum density after FFT (fast Fourier transform) of an R-R interval (Figure 1).

It is important that the driver monitoring system must not restrict or interfere with the driver’s action [26]. Table 1 shows the general and traditional methods of measuring heart rate based on Reference [27].

According to Table 1, general and traditional methods of measuring heartbeat restrict or interfere with the driver’s action. Therefore, these methods are not inadequate to measure heartbeat in a vehicle, and a nonwearable-type monitoring system is desirable in spite of the fact that the reliability of the acquired data is inferior to that of wearable-type systems. Such driver monitoring systems must be able to detect the driver’s state of alertness correctly without making contact with the driver. However, certain wearable-type systems, such as clothes-type or ring-type systems, do not restrict the driver’s action. Therefore, certain wearable-type driver monitoring systems are in use. In Japan, a law subsidizing the cost of acquiring equipment was certified by the Ministry of Land, Infrastructure, Transport, and Tourism, in order to incentivize the installation of driver monitoring systems to prevent drivers from overworking themselves and avoid serious accidents caused by drivers falling asleep at the wheel [28].

In this review, examples of heartbeat-monitoring systems are introduced, and future prospects for the implementation of such devices are presented. The requirements for heart-rate detection systems in a driver-piloted vehicle are introduced, and the methods for detecting the driver’s heart rate in vehicles are reviewed. In addition, the prospects for developing heart-rate detection devices are also explained, with consideration to the widespread adoption of smartphones and smartwatches at present. This review focuses on automotive application heart rate monitoring systems and addresses the widespread use of smartphones and smartwatches, as well as discusses heart rate monitoring systems for the autonomous driving era. These are the major differences from the previous review by Sidikova et al. [26]

Here, the method for the literature review is as follows:

- In advance, based on the experiences of the author, who was an engineer of an automotive manufacturer, and prior discussions with the manufacturer’s engineers, the type and measuring method of the heart rate detection system are summarized.
- We used google scholar to search for papers on the types and measurement methods we had identified and selected relatively new papers as the target of our investigation. In some cases, however, the papers were not disclosed due to patents or were not published as a paper because the heartbeat monitoring system is more about development than research. In such cases, the survey was conducted on the Web, and relatively new content was selected for the survey.

The survey is not based on any specific method or quantitative technique and is somewhat intuitive, but we believe that we have covered most of the subjects based on empirical data.

The remainder of this paper is organized as follows. Section 2 includes a review of research and development trends. Section 3 discusses the significance of a heart rate monitoring system in the autonomous driving era. Conclusions are presented in Section 4.

## 2. Trends in Research and Development

From the results of the survey conducted for this review, trends in research and development on heartbeat monitoring are categorized into two types:

- Algorithms for high accuracy and steady measurement of heartbeat;
- In-vehicle heartbeat measuring systems focused on driving.

It is prohibitive to cover the totality of research and development on the stated topics; therefore, we cover typical examples in this review.

### 2.1. Research Trends in Algorithm Development

Ye et al. considered the spectrogram of the received Doppler radar signal as a linear mixture of source signals such as heartbeat, respiration, and body motion, extracting only the heartbeat component by non-negative matrix factorization and then using sparse vector regeneration to achieve accurate estimations of heart rate [29]. However, the evaluation was not performed for a driver in a moving vehicle.

Izumi adopted a signal processing algorithm combined with time-frequency analysis and template matching, using a microwave Doppler sensor as a method for heartbeat detection [22]. The heartbeat was detected even when body motion noise was superimposed; this algorithm enabled the measurement of a steady heartbeat for a driver in a moving vehicle. The study included eleven participants and measured the heartbeat in drivers in a vehicle moving at 50 km/h, achieving an RMSE of 13.11 ms.

Project Olive improved the detection accuracy based on a combination of differential methods and frequency analysis [30]. The project achieved ambivalent rapidity and high accuracy for detecting R-wave peaks by combining multi-resolution analysis with fast processing of data signals and variants of the Hilbert transform and the Hamilton and Tompkins algorithms. Multi-resolution analysis enables the selection of only the signal containing important information; therefore, the R-wave peak can be detected after reducing the factors depending on a driver’s personality, such as sex, age, the state of cardiovascular functioning, and other noise effects, using an optimization algorithm based on the Hilbert transform and primary differential.

### 2.2. Research Trends in Heartbeat-Measuring Systems Applied to Drivers in Vehicles

In-vehicle heartbeat-measuring systems are classified into two types: the wearable type, in which a person wears a wristwatch-type wearable device, and the nonwearable type, in which the driver’s condition is measured at the steering wheel, seat, or other places the driver touches while driving.

The wearable and nonwearable systems can be roughly summarized as shown in Table 2.

#### 2.2.1. Measurement by Wearable-Type Devices

Currently, there are many watch-type wearable devices, and devices such as the Apple Watch [31], Empatica E4 [32], Garmin Watch [33], Fitbit surge [34], and Jawbone UP3 [35] are sold on the market. The approaches used for measuring heartbeat in these devices are almost the same. The blood-flow to the wrist increases with the heartbeat, and the green light used in the device is well absorbed. In contrast, the green light is less likely to be absorbed at the time between heartbeats. By making use of this principle, the heartbeat is measured by flashing an LED light behind the watch several hundred times per second [36]. In addition, such devices can be linked to smartphones to share data. As for accuracy, Falter et al. show that, in patients with cardiovascular disease, the Apple Watch measures HR with clinically acceptable accuracy during exercise [37]. Schuurmans et al. also show that the Empatica E4 is a practical and valid tool for research on HR and HRV under non-movement conditions [38]. Gillinov et al. shows that the correlation between the HR of an electrocardiograph and that of a wrist-type monitoring system during aerobic exercise is between 0.67 and 0.996 [39].

As these wristwatch-type wearable devices are released to the market and worn by an increasing number of people, the trend of in-car measurement devices appears to be changing. The wristwatch-type devices can measure the heartbeat naturally, without restricting the driver’s motion. If the only purpose of a monitoring device is to measure the heartbeat, it will obviate the need to install a nonwearable driver-monitoring system in a vehicle (which entails an extra cost). The Ford motor company stopped developing its seat-type heartbeat-monitoring sensor in 2015. The company stated that the reason for this decision was the advent of wearable devices, such as the Apple Watch, Empatica E4, Garmin Watch, Fitbit surge, and Jawbone UP3 [40]. Therefore, the emergence of wearable devices has transformed the use of automotive heartbeat sensors.

As far as we could ascertain, the major type of in-vehicle driver-monitoring system at present is the camera type, and there are not many examples of steering-wheel or seat-type systems being developed. One possible reason for this is that Ford has stopped developing heart-rate monitoring sensors built into seats. An additional factor might be that the advent of wearable devices opens up the possibility of segregating biometric information into information that can be obtained by wearable devices and information that cannot be obtained by wearable devices but can be obtained by cameras.

The main function of wristwatch-type devices such as the Apple Watch is that of a watch, and heart-rate sensing is a secondary function. However, there is a device on the market which has the purpose of managing a driver’s health [41]. This device can also be linked to smartphones to share data. They seem to be selling their services rather than their devices. In addition to this device, there are many other devices in which the heart rate sensor is the only function and can link to smartphones to share data, in consideration of the recent health trends [42,43,44]. These devices are relatively inexpensive, approximately lower than US$150. Since single board computers such as Arduino [45] and raspberry pi [46] are widely available and inexpensive, people who are good at or knowledgeable about digital fabrication and electronics can build these devices themselves.

For example, one case study shows how to develop a wearable heart rate monitor for Arduino [47]. An Arduino Nano R3 is used in this case study. The Arduino Nano R3 has been combined with a generic nRF24 Module together a Adafruit NeoPixel Ring [48] to provide visual feedback on heart rate. This DIY wearable heart rate monitor cannot be linked to a smartphone, but by attaching a Bluetooth or Wifi module to the Arduino Nano R3, this monitor may be able to work with a smartphone. The DFRobot heart rate sensor is a commercial and thumb-sized heart rate monitor designed for Auduino microcontrollers [49]. The sensor has two holes that can be used to attach to the belt and can be wrapped on the finger, wrist, earlobe or other areas where it has contact with the skin. The interface of this sensor is adapted to allow plug-and-play to ease the barrier of usage, and this sensor can compatible with Arduino, Raspberry Pi, intel edison or other microcontrollers; therefore, this sensor can also link to a smartphone. In any case, wristwatch-type heartbeat monitoring devices, whether commercial wristwatch-type devices or DIY devices, are readily available. As in these cases, there are several previous studies in which DIY heartbeat monitoring systems have been used, and DIY heartbeat monitoring systems seem to be becoming more common these days [50,51,52].

There are devices that attach to the finger like a ring and measure the pulse wave [53,54,55], and devices that hang around the neck and measure the pulse wave after attaching an ear clip sensor to the earlobe [56]. The latter types of devices have the characteristic of obtaining pulse data that are different for each individual via the blood vessels in the earlobe; such devices can detect the state of a driver’s heart with high accuracy after analyzing the acquired data.

In addition to wristwatch-type, ring-type, and necklace-type devices, there are shirt-type wearable devices. These have electrodes in the shirt that can measure pulse waves when the shirt is worn [57]. These types of devices use a flexible, stretchable, and breathable electrode material coated with a conductive polymer (PEDOT: a composite material of PSS (poly(3,4-ethylene dioxythiophene)) polystyrene sulfonate) and a nanofiber textile. The electrode material has excellent flexibility, elasticity, and breathability. This material is also highly hydrophilic, so that it absorbs perspiration and steam, making it easier to adapt to the skin, and it can measure a stable electrocardiogram comparable to that of medical electrodes without the use of electrolyte paste. Because the electrodes are used inside the shirt, the heart rate and pulse wave can be acquired by wearing a shirt [57].

Such a wearable monitoring system is not acceptable to non-professional drivers because many non-professional drivers would not agree to wear that while driving. However, such a wearable monitoring system could be rented to professional drivers as a uniform, making them less reluctant to wear it because they need to wear because they continue to wear it and get to feel “this is uniform, therefore I must wear” and allowing their health status to be acquired while they are at work. In addition, it would be easier to manage drivers’ labor if the data were uploaded to the cloud server.

#### 2.2.2. Measurement by Nonwearable-Type Devices

Nonwearable-type heartbeat monitoring systems are mainly categorized into steering-type, seat-type, seat-belt-type, portable device-type, and camera-type systems.

As an example of these types, we discuss a system developed by Yanagidaira and Yasushi [58]. This system’s design includes an ECG transmitter and receiver. The transmitter and receiver are wirelessly connected to each other. The transmitter consists of a steering cover, which operates as an electrode for detecting the ECG, and a steering sensor, which consists of a transmitting ECG unit. The transmitting unit amplifies the potential difference, which is caused when the driver grasps the steering; after the potential difference is filtered, the frequency band of the ECG signal is filtered. This signal is converted to a digital transmission signal in the RS232C format and sent wirelessly to the receiver. The receiving unit demodulates the received signal and sends it to the PC. The driver-state estimation program extracts R waves from the ECG sent by the receiving unit and calculates the heartbeat, HF, and the frequency of breathing. The level of sleepiness of the driver is estimated from these measures. The level of sleepiness is evaluated on a scale ranging from 0 (not at all) to 6 (maximum), based on the amount of heart-rate reduction. In addition, the level of sleepiness is affected when the HF increases continuously over a certain period. If the signals cannot be detected because the driver has grasped the steering with only one hand, the missing data are complemented and estimated based on the already acquired data. The signals are processed in such a way that a driver’s state is well estimated even if the driver uses one hand for half of the total driving time.

Nakagawa et al. introduced a method in which a pulse wave was detected by grasping the electrodes equipped onto the steering wheel [59]. The electrode was an acrylonitrile butadiene styrene (ABS) resin plated with chrome. A pulse wave was detected by a photoelectric pulse wave sensor set on the steering. The LEDs and photodetectors were placed where the driver’s palm touched the steering wheel when he gripped it, and the pulse wave was calculated by the reflected LED light emitted to the driver’s skin. A blood oxygen saturation (SpO_2_ sensor is often used as a pulse wave sensor; however, it is not applicable in this context. An SpO_2_ sensor works with red-light and near-infrared-light LEDs that are transparent to the skin; however, the use of an SpO_2_ sensor requires that the driver’s finger or hand must be placed between the LED and the photodetector, thus restricting the driver’s motion. Thus, in the case of Nakagawa et al., a green reflective pulse wave sensor was used.

**Figure 2:**
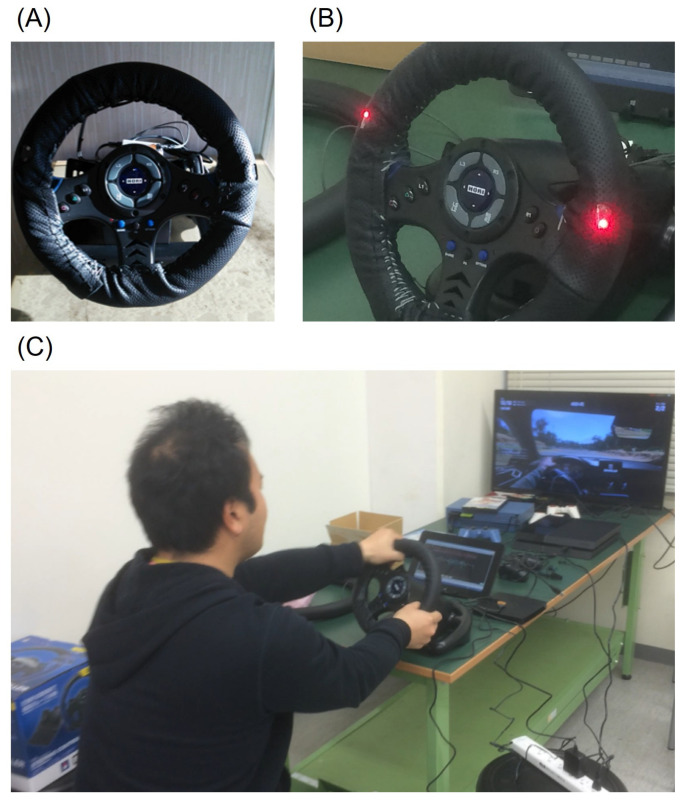
Steering-type measurement system developed by Arakawa et al.: (**A**) appearance of the system; (**B**) transmitter and receiver LEDs visible on the spoke of the steering wheel; (**C**) demonstration of the operation of the system while playing a driving game.

**Figure 3:**
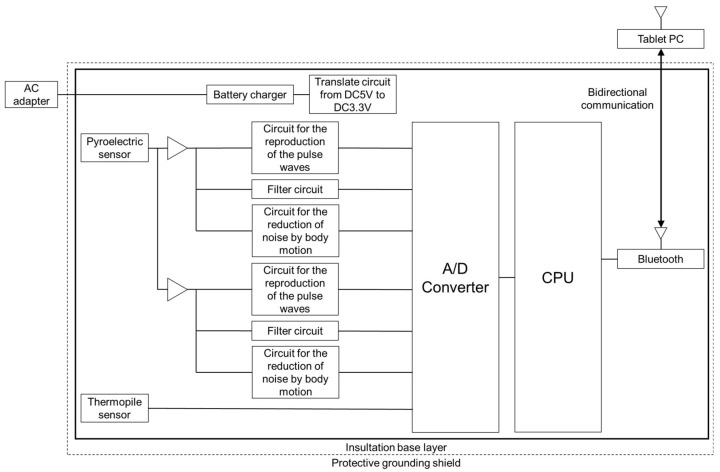
The system block diagram of the steering-type measurement system developed by Arakawa et al.

Arakawa et al. developed a steering-type measurement system equipped with a transmitter and a receiver for an LED on the spoke of the steering wheel (Figure 2) [60]. This system can detect heartbeats based on the capacitive pulse method, which detects heartbeats by considering the amount of blood passing through the tissue per pulse wave. This system can stably measure pulse waves regardless of the driver’s movement while driving. In the context of mass production, this system uses red LEDs because the costs are lower than for green LEDs. As mentioned earlier, this system can measure pulse waves stably regardless of the driver’s movement while driving, but the system does require that the driver holds the steering wheel firmly while driving, whether driving one-handed or two-handed, which slightly restricts the driver’s movement. The system block diagram is shown in Figure 3.

The steering-type system developed by Essers et al. [61] is similar to the system described by Arakawa et al. The system includes an infrared sensor built into the steering wheel. The driver’s facial expression can be detected by the sensor; this detection is coupled with data obtained through sensors that can detect the driver’s hands on the steering wheel. The system estimates the driver’s status by combining the heartbeat with other evaluation indices. Essers et al. also stated the importance of a redundancy system that can ensure a stable system.

However, the steering-type system is useless if the SAE’s levels of driving automation level 4 or 5 [62] will be realized because the driver needs to be holding onto the steering wheel. Thus, the steering-type system is useful under the SAE’s levels of driving automation lower than level 3 [62].

**Figure 4:**
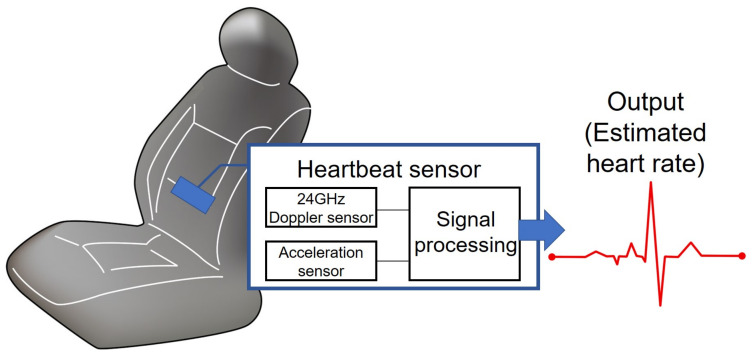
The outline of Mitani’s system.

Mitani [63] offered an example of a heartbeat-monitoring system using detection through the surface of the seat. The pulse sensor is built into the driver’s seat, and the pulse rate is estimated by digital signal processing using the signal detected by a radio sensor. In addition, an acceleration sensor detects and discriminates the shocks caused by vehicle vibration, thereby preventing the output of an incorrect pulse rate. The device is controlled by SPI communication to set the registers of the microwave sensor and accelerometer and obtain acceleration data from the accelerometer. The acceleration data are used to detect disturbances caused by impacts to the sensors. The sensor can be connected to an external unit through CAN communication and is sufficiently compact to be embedded in the seat. However, when the sensor is embedded in the seat, the distance variation between the body surface and the sensor, as well as the distance variation between the sensor and the surrounding metal objects becomes a major disturbance factor. Figure 4 shows the outline of Mitani’s system.

Furthermore, in addition to the pulsations being observed, the body surface is also subject to disturbances such as spontaneous body movements caused by breathing, limb and head movements, and impact body movements caused by vehicle vibration during driving. In that study, three main technologies were developed to solve these problems:

1. Correction of the offset shift of I-Q Lissajous due to changes in the positional relationship with the surrounding metal objects.
2. Synchronous detection for detecting pulsation-period signals buried in external disturbances. A model pulse wave signal was generated by applying the feedback of the estimated pulse wave number. The difference between this signal and the pulsation signal after the adaptive filter processing was recorded. The adaptive filter coefficients were updated so that the mean-square deviation was minimized.
3. Removal of periodic artifacts, such as respiratory harmonics. The respiratory component was extracted from the Doppler angular velocity signal, and the harmonics were estimated. The signal was passed through an adaptive filter, the difference from the Doppler angular velocity signal was recorded, and the coefficients of the adaptive filter were updated to minimize the mean-square deviation.

In the case study, the system was evaluated on a real car. With regard to the RMS of the pulse rate, estimates of 6 people in a resting state and 13 people driving in an urban area were procured. The RMS error in the resting state was within 5 bpm, and in the urban area, it was generally within 10 bpm. However, the error for one of the subjects was large, and it was assumed that this was because the body surface of the measurement site was compressed by the seating posture, making it difficult to detect the pulsation component and preventing a correct measurement.

Mitani’s system is supposed to be pre-installed when buying a car; however, there are some systems that can be installed after purchasing a car, making it more convenient for the user. Kojima et al. developed a vehicle seat in which an air pack sensor can be installed after the driver buys a car [64]. This seat can detect biological signals such as pulse waves in the trunk and breathing through the driver’s back. The signal from the air pack was filtered to isolate the pulse wave signal from the trunk. The isolated signals were defined as air pack pulse waves (AP-PW). The output signal of the sensor that detects fluctuations in the air pressure of the air cushion is filtered at a predetermined frequency to extract the carrier wave containing the arterial pulse wave component (first filtering), and the signal wave that is obtained by this filtering is registered by a detector. The signal wave of the pulse wave component registered by the detector is filtered within the range of the cutoff frequency on the add/drop side that is higher than the frequency of the respiration component, and the cutoff frequency on the upper side that is based on the frequency of the second harmonic component (second filtering). The first filtering extracts the carrier wave of the arterial pulse wave component, and the second filtering detects the pulse wave component, including the second harmonic component, by filtering with the frequency of the second harmonic component as a reference. In this way, the pulse wave can be captured in the final signal wave, indicating that the biological information has been reliably captured.

Delta Kogyo Co., Ltd. applied this principle and mass-produced their product as the “Sleep Buster” [65]. This product was eligible to receive support as part of a project promoting support for accident prevention measures [66]. The Fraunhofer Institute for Photonic Microsystems (IPMS) has developed an ECG that operates from within a driver’s seat [67].

There are a few cases in which a driver’s pulse wave is detected via the seat belt. HARKEN is developing a system that can detect the heartbeat and breathing of a driver via sensors attached to the seat cover and seat belt [68]. This system can calculate the parameters of the heart rate and breathing after the noises and artifacts (vibration and body motion) are filtered and canceled while driving.

**Figure 5:**
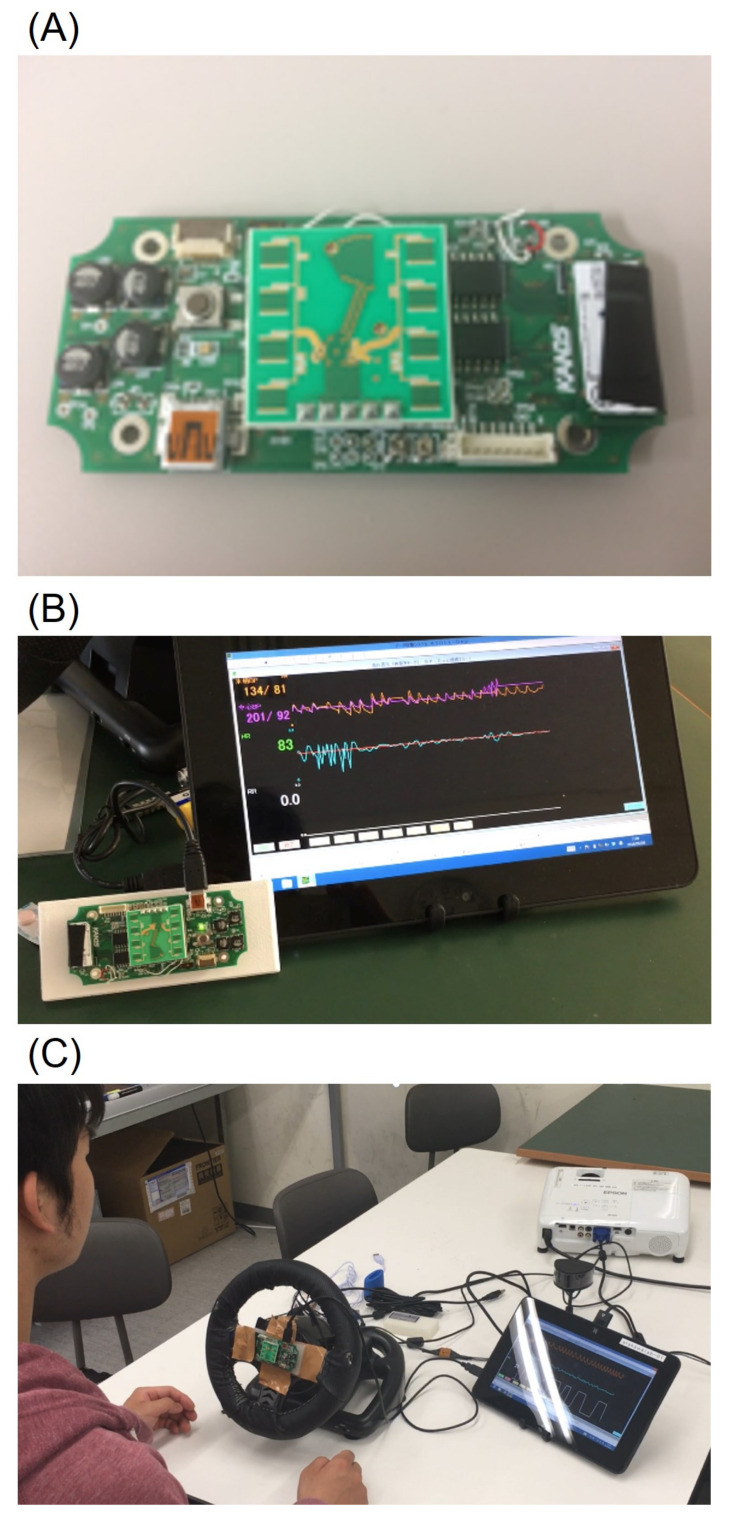
A portable device-type system developed by Arakawa et al.: (**A**) developed portable device-type system (**B**) appearance of the system and interface that shows the current blood pressure; (**C**) demonstration of the operation of the system.

**Figure 6:**
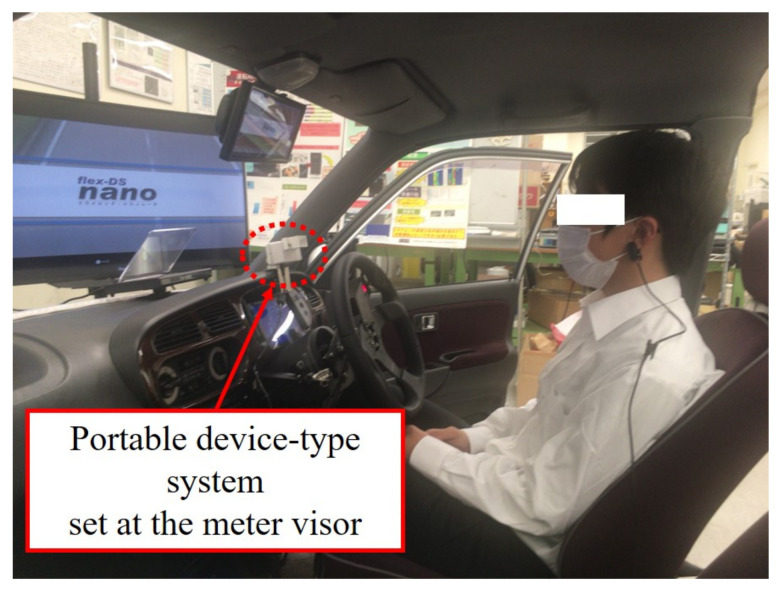
Portable device-type system set at the meter visor.

There are also some cases of portable device-type, which are intended to be set at the meter visor and around the center console. Texas instruments developed the AWR1642 sensor, and this sensor is mounted above the rearview mirror and has two transmitters and four receivers [69]. They explained that this sensor can detect the heartbeat and occupancy detection of four occupants simultaneously, and also enable additional features beyond occupant detection, such as basic classification of occupants as an adult or child [69]. Arakawa et al. developed a portable device-type system, which is intended to be set at the meter visor [70,71] (Figure 5). An ultrasonic doppler is used for this system. This system is for in-vehicle and can measure blood pressure monitor continuously. This system is intended to set at the meter visor as in Figure 6.

In recent years, cameras have become more inexpensive, and their performance has improved, and with the rise of smartphones, research and development of camera-type systems has progressed. Some mass-produced cars are equipped with in-vehicle driver monitoring cameras, and their functions are limited to detect signs of driver distraction or drowsiness [72,73,74,75,76]. For example, Subaru’s Driver Monitoring System (DMS) has functions in which it alerts the driver when it detects signs of his/her distraction or drowsiness. When DMS detects the driver’s eyes shutting or looking aside for an extended period of time, it determines the driver is distracted or drowsy, and alerts the driver via audio and visual warnings on the meter and multifunction display [72]. In addition, DMS recognizes up to five pre-registered drivers to provide the following customized driver settings [72]. It is said that Tesla is starting to use the camera above the rearview mirror in the Model 3 and Model Y to help make sure people pay attention to the road while using autopilot because regulators and safety experts have begged Tesla to add better driver monitoring [77].

However, few devices have emerged that specialize in detecting heart rate from images captured by a camera and applying it to automotive applications. Countermeasures have to be implemented to deal with interference from external light inside the vehicle cabin when applying the system to vehicles. In what follows, we limit our discussion to examples of systems that have been developed only for use in laboratories and offices.

Sakamaki et al. developed a system that can estimate heartbeat using images captured by a camera based on the brightness of a person’s skin [78]. In this system, the target area to be measured is divided into n areas, the average brightness is calculated for each area, and the peak brightness is calculated after the application of a discrete Fourier transform. Next, the frequencies of the detected peaks are sorted in descending order of the peak heights. A function termed “$g_{i}$” can acquire the index number after sorting in the *i*-th area. A weighted count-up is performed using $g_{i}$, and the heartbeat is estimated based on this result. Okada et al. attempted to separate the facial image into melanin pigment, hemoglobin pigment, and shading intensity components by independent component analysis (ICA) and estimated the pulse wave based on the changes in these components over time [79]. Kwon [80] appears to have performed a similar exercise to the case of Okada et al. There are some reports of heartbeat detection based on thermographic information; however, thermography seems to be mainly used for detecting breathing information [81].

Rizmill [82] features the ability to detect video pulse waves using a general-purpose web camera, which enables health management at home as well as infant monitoring. Their product also provides services such as broadcasting the visualized pulse wave data before and after a golf shot is taken, as viewer entertainment. Therefore, it is expected that cameras will increasingly be used to detect pulse waves as part of a service strategy for healthcare.

With the rise of smartphones, the use of sensors in commodity devices is being explored. The point is how to improve healthcare using smartphones [83], and Google Fit, the application of the Google smartphone, Pixel, developed by Google, enables the measurement of the number of heartbeats and breathing [84,85]. Considering the connection between smartphones and smartwatches and that almost all drivers always bring their smartphones into the cabin with them, Google might choose to exploit the opportunity for persons to manage their health under a large range of circumstances, including when driving a vehicle. In particular, when envisioned for in-vehicle use, a gadget that allows the driver to wear a smartphone in the car to monitor pulse waves and other conditions could provide value and convenience to the driver. For smartphones, HRV4Training [86] seems to be a useful application, such as Google Fit. This application can be used on both Android OS and iOS. There are some studies using HRV4Training [87,88]. The appeal point of HRV4Training is that the external sensor is not needed and HRV can be measured using the camera and the flash of the smartphone [89]. However, it is not possible to measure heart rate in a non-contact manner because HRV can only be measured by touching the camera with a finger.

**Figure 7:**
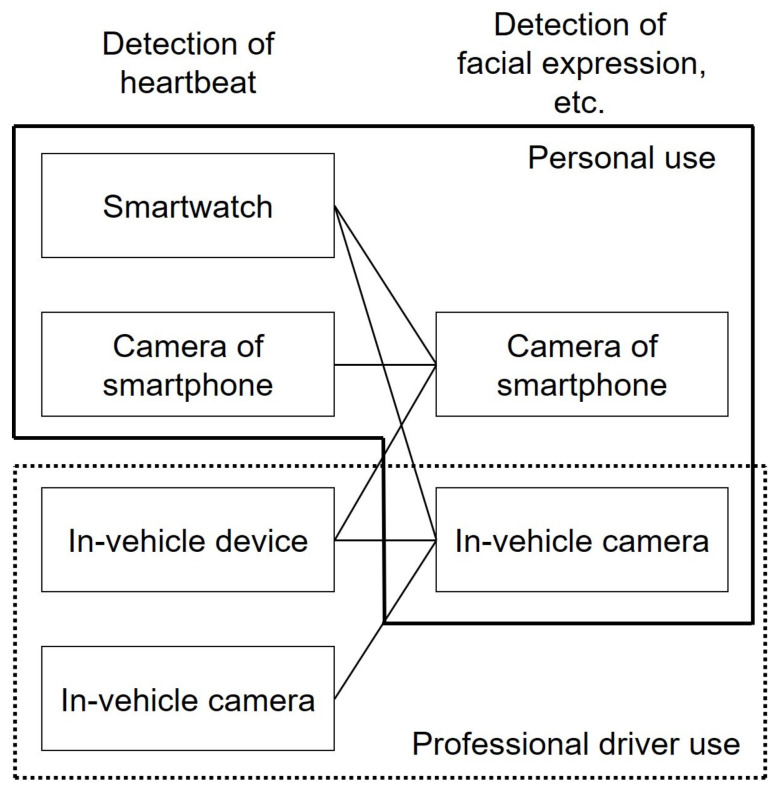
Segregation of the device for detection of heartbeat and expression. The lines connected from each block of “Detection of heartbeat” to each block of “Detection of facial expression, etc.” show the possible combination for adapting in-vehicle use.

There is also a method that extracts the pulse signal of blood flow using a chrominance-based method instead of RGB images for images taken with a smartphone and obtains the R-R interval by using a denoising method (CWT-max) with wavelet transform and inverse wavelet transform [90]. The afore-going discussion makes it clear that the rising adoption of smartphones cannot be ignored. Given the fact that the penetration rate of smartphones is said to be 78% [91], and that ownership of smartphones has become common, coupled with the increasing connectivity of vehicles [92], it is our impression that the number of methods for detecting heartbeats using smartphone cameras is increasing [92]. Unlike in-vehicle systems, when using a smartphone, one can carry the device and application with which one is familiar even when riding in a vehicle other than one’s own, such as a rental car. Smartphone-based heart-rate detection systems can be easily used by simply attaching a smartphone to the system. However, because smartphones cannot be forced to provide information to the cloud, it may be necessary to use a company-issued smartphone when managing the operation of laborers, whereas drivers may not necessarily use a company-issued smartphone. However, it would not always be the case that drivers are forced to use company-issued smartphones. It is also possible that drivers intentionally modify or upgrade their personal smartphone applications. Therefore, for labor management, an in-vehicle heart rate detection device may be more desirable to prevent modification or recovery. On the other hand, the heart rate and pulse wave detected by a camera are not equivalent to the values obtained by directly evaluating pulse waves and heart rate signals, so it is important to ensure accuracy. From the afore-going discussion, the following segregation of heart rate detection can be considered (Figure 7):

In other words, smartwatches and smartphone-based devices are expected to be used for personal purposes because they can be attached and detached at will, and there is no obligation to wear them. On the other hand, if labor-management considerations dictate that the measurement of driver status should be compulsory, we should consider the possibility of segregation into in-vehicle devices and in-vehicle cameras that cannot be attached or detached at will.

However, in the case of smartwatches and smartphone-based devices, there is a concern that measurement may not be possible when the device is not in possession of the driver or when it is being charged. In addition, it is assumed that the device will always be connected to the in-vehicle system, and robustness of connectivity will be required. As it is impossible to predict when and if a sudden change in physical condition will occur, it is necessary to have a sensing environment active at all times. In addition, because there are very few published evaluations of in-vehicle detection using these devices, the authenticity and reliability of detection accuracy when applied to in-vehicle applications are not yet guaranteed. Therefore, until the above-mentioned issues of recharging, the robustness of connectivity (e.g., until 5G becomes mainstream), and reliability of detection accuracy in in-vehicle applications are ensured, detection methods using in-vehicle devices and in-vehicle cameras will remain the mainstream methods for monitoring driver status. We can expect to see the emergence of devices that will be able to provide health services and the detection of abnormal driving behavior simply by driving vehicles equipped with such devices as usual.

Considering the above, the optimal combination can be summarized as follows (Figure 7):

- For the time being, in-vehicle devices or in-vehicle cameras are used for the detection of a heartbeat, and in-vehicle cameras are used for the detection of the driver’s facial expression.
- Smartwatches and the camera of the smartphone are used for detection of heartbeat, and the camera of the smartphone is also used for detection of driver’s facial expression after some issues such as recharging and connectivity are solved perfectly.
- However, as for the professional driver, in-vehicle devices or in-vehicle cameras are used for the detection of a heartbeat, and in-vehicle cameras are used for the detection of the driver’s facial expression because they are obliged from the viewpoint of labor management.

The comparison of wearable-type and nonwearable-type heartbeat measuring methods in this review is shown in Table 3, Table 4, Table 5 and Table 6. From Table 3, Table 4 and Table 5, in many cases, the accuracy of the system developed by the manufacturer is not stated. Therefore, if we use the heartbeat measuring system by the manufacturer, it is important to evaluate the accuracy at first. Many of the systems presented in the paper have not also been sufficiently evaluated for accuracy, and there are some cases in which the accuracy seems to be evaluated from the concordance of the waveshape of ECG and the pulse acquired by the system. However, the wearable type seems to be more accurate than the nonwearable type.

Table 6 shows the advantages and disadvantages of each measuring method. There seem to be no large differences from each measuring methods of each type of system. In particular, for nonwearable types, the choice of measuring methods will be left to the design issues of the vehicle, such as cost and mounting location.

## 3. Proposal for the Autonomous Driving Era

We have discussed how heart rate detection monitors should be used in current or near-future automobiles, especially less than SAE’s standards for autonomous driving level 3. For the vehicle of autonomous level 0-3, the main purpose of heart rate detection monitors is to detect the driver’s state such as attention, workload, or arousal level. However, we should consider the autonomous driving levels 4 and 5, and Operator 4.0.

Operator 4.0 focuses on treating automation as a further enhancement of the human’s physical, sensorial and cognitive capabilities by means of human cyber-physical system integration [93]. This may coincide with autonomous driving levels 4 and 5 in that the driver does not need to operate the vehicle, and the vehicle takes the place of the driver in spite of the driver’s state. Systems in autonomous driving levels 4 and 5 may shift from detecting the driver’s state to the coordination between the driver and the autonomous vehicle.

For example, Vanderhargen et al. show the synchronization between dynamic events with heartbeats and its impact on non-conscious errors in the control of dynamic events [94]. In research by Dey et al., provided with real-time heart rate feedback, participants felt the presence of the collaborator more and felt that they understood their collaborator’s emotional state more. Dey et al. also found that heart rate feedback made participants feel more dominant when performing the task [95]. This research is not aimed at automobiles, but I think it can be replaced by autonomous driving. For example, two autonomous vehicles (driven by friends or family members) heading to the same destination could be connected to each other, and each driver could be able to sense the other’s feelings by communicating the heartbeat of each driver and providing feedback on the other’s heartbeat.

In terms of the relationship between the driver and the self-driving car, it may be possible to reflect the driver’s heartbeat in the control of the self-driving car. Vanderhaegen proposes a heuristic-based prospective method to discover possible conflicts of shared control between humans and autonomous systems and applied this method to an autonomous driving case study [96]. As a result, he found that the heuristic-based method can detect possible conflicting decisions or sources of conflicts between humans and machines [96]. As for the autonomous vehicle, heartbeat detection may replace this heuristic-based prospective method. For example, the driver’s heartbeat during autonomous driving can be detected continuously, and if a change in a heartbeat is observed during certain vehicle control and the driver is thought to be feeling nervous or anxious, the vehicle control can be made milder to alleviate the driver’s nervousness or anxiety. It could be that the control is fine-tuned so that the driver’s heartbeat is stabilized while the driver and vehicle are always in a coordinated state. The above is just an idea, but there may be many other useful methods.

As described above, a heart rate monitoring system will not be used to detect the heart rate and provide information to the driver as in conventional automobiles but will be used as a means of cooperation between the driver and the automobile in self-driving cars. However, the segregation of heart rate detection might remain as in Figure 7 for a while.

## 4. Conclusions

In this review, we discussed the current status of heart-rate detection systems in vehicles. The emergence of wearable devices such as the Apple Watch is considered to be a turning point in the application of heart-rate detection while driving vehicles. There are no recent examples of systems or devices that detect heart-rate signals or estimate the driver’s state based on the heart rate alone. Wearable devices such as wristwatches, which can be worn easily without restraining the driver, are responsible for heart-rate detection alone. In addition, with the rise of smartphones in recent years, devices with cameras have become portable, which is also considered a major development. On the other hand, whether or not to make it compulsory to acquire a driver’s status and whether or not to acquire the status in environments other than the familiar vehicle environment will have a major impact on the type of device to be used. On the other hand, for occupational drivers for whom the need to obtain the data might be made compulsory from the viewpoint of labor management, it will be important to distinguish between the two approaches applicable to in-vehicle use. The current smartwatch and smartphone-based systems have various problems associated with them, and there are barriers to their practical use in vehicles. Therefore, for the time being, detection methods using in-vehicle devices and in-vehicle cameras are expected to remain in the mainstream application, and the emergence of devices that can detect health conditions and abnormalities simply by driving in the usual manner is expected.

In addition, we argued about the future prospect of the heart rate monitoring system especially under the SAE’s standards for autonomous driving level 4 and 5: the heart rate monitoring system will not be used to detect the heart rate and provide information to the driver as in conventional automobiles but would be used as a means of cooperation between the driver and the automobile in self-driving cars.

In this review, we discussed and argued the current and future prospect of the in-vehicle heart rate monitoring system. It is necessary to proceed with the design and development considering the possibility of transition from the current driver monitoring application to the driver–vehicle coordination in anticipation of future autonomous driving, which is another point we would like to insist on in this paper. By looking at the current and future technological trends in automated driving, as well as the development trends of heartbeat detection and monitoring systems, it is necessary to consider the appropriate hardware and applications.

## Figures and Tables

**Table 1:** Measuring heart rate methods and weak points of the general and traditional method.

Method	Technique	Device	Weak Point
ECG (Electrocar- diogram)	Measures the electrical pulses generated by the body during each cardiac cycle	Electrodes	Difficult to position correctly, affected by body movement, electrodes become loose when the body is sweaty
Sphygmomano- meter	A method for measuring changes in arterial pressure that vary with heart pulsation	Sphygmomano- meter	Affected by body movement
Cardiogram	Measure the sound generated by the pulsation of the heart	Finger, stethoscope, microphone	Affected by hand and finger movement
Photoelectric pulse	Near-infrared light is applied to the skin surface and the reflected light is received by a photodiode or other device	Smartwatch	Affected by body movement, touching condition of fingers, skins, etc.

**Table 2:** Wearable and nonwearable systems.

Type of System	Measuring Methods
Wearable-type heartbeat measuring system	Wristwatch-type
	Ring-type
	Necklace-type
	Shirt-type
Nonwearable-type heartbeat measuring system	Steering-type
	Seat-type
	Seatbelt-type
	Portable device-type
	Camera-type

**Table 3:** Comparison table of wearable-type heartbeat measuring methods in this review. NA means that no description of accuracy can be found.

Measuring Methods	Developers/ Authors	Year	Technology	Accuracy Compared to ECG
Wristwatch- type	Apple [31] Empatica [32] Garmin [33] Fitbit [34] Jawbone [35]	2015	Flashing a green LED light behind the watch several hundred times per second	Acceptable, 0.67 ≤r≤ 0.996 (during aerobic exercise)
Ring-type	Jung et al. [53]	2007	Flashing an LED light inside the ring several hundred times per second	NA
	Oura [54]	2016		R_2_ = 0.996 for resting HR and R_2_ = 0.980 for HRV
	TheTOUCH [55]	2016		NA
Necklace-type	Fujitsu [56]	2015	Wear the ear clip sensor on the ear to acquire vital data	NA
Shirt-type	Kasai et al. [57]	2015	Measure pulse waves by the electrode in the shirt	NA

**Table 4:** Comparison table of nonwearable-type heartbeat measuring methods in this review. NA means that no description of accuracy can be found.

Measuring Methods	Developers/ Authors	Year	Technology	Accuracy Compared to ECG
Steering- type	Yanagidaira and Yasushi [58]	2003	Measure by electrodes equipped onto the steering wheel	There is a correlation between HR change and subjective value of sleepness
	Nakagawa et al. [59]	2016		NA
	Arakawa et al. [60]	2018	Flashing an LED light equipped onto the steering wheel	The same level of performance as a commercial electronic sphygmomano- meter
Seat-type	Mitani [63]	2019	Microwaves irradiated to the driver’s body and Doppler signals of the reflected waves	RMS error while driving is between 5 and 10 bpm
	Murata et al. [64]	2011	Body-trunk plethysmogram signal detected by air-pack	NA(some say it is over 90%)
	Delta Kogyo [65]	2012		
Seatbelt- type	HARKEN [68]	2014	Sensor embedded in the seat cover and the seatbelt	NA
Portable device-type	Texas instruments [69]	2019	Using Texas instruments mmWave sensors	High accuracy
	Arakawa et al. [70,71]	2017	Infrared radar irradiated to the driver’s body and Doppler signals of the reflected waves	Not high accuracy due to body movement

**Table 5:** Comparison table of nonwearable-type heartbeat measuring methods in this review (continued). NA means that no description of accuracy can be found.

Measuring Methods	Developers/ Authors	Year	Technology	Accuracy Compared to EEG
Camera- type	Sakamaki and Fujita [78]	2020	Signal processing of person’s skin image	BPM difference is less than 3 bpm
	Okada et al. [79]	2018	Change in the average pixel value of the hemoglobin component images obtained using the skin pigment separation on the RGB pixel values of facial images	NA (only the accuracy of facial expression classification was evaluated)
	Kwon et al. [80]	2012	Facial color change took by a smartphone’s camera	NA
	Sun et al. [81]	2018	Facial color change took by RGB camera	r=0.96
	Google [85]	2014	Pulsewave detected by smartphone’s camera	NA
	HRV4Training [86,87,88]	2015		MAPE, median and IQR of rMSSD is 4.10, 2.76 and 3.74, respectively
	Huang [90]	2016	Facial color change took by smartphone’s camera	The mean of absolute errors of HRV metrics is 3.53 ms

**Table 6:** Advantages and disadvantages of heartbeat measuring methods in this review.

Type of System	Measuring Methods	Advantages	Disadvantages
Wearable- type	Wristwatch-type	Accuracy seems to be guaranteed, easy to wear, many people have them and easy-to-use	Cannot measure if forget to wear, there are some unsolved problems such as recharging and the robustness of connectivity
	Ring-type	Accuracy seems to be guaranteed, easy to wear	
	Necklace-type		
	Shirt-type		Discomfort to wear
Nonwearable- type	Steering-type	Not restrict the driver’s behavior, can measure at any time in spite of driver’s willing	Need to be installed at the time of purchase or after purchase the vehicle,
	Seat-type		
	Seatbelt-type		
	Portable device-type		
	Camera-type		Accuracy in the cabin has not been fully evaluated
